# Evaluating the integration of tuberculosis screening and contact investigation in tuberculosis clinics in Ethiopia: A mixed method study

**DOI:** 10.1371/journal.pone.0241977

**Published:** 2020-11-19

**Authors:** L. Ketema, Z. G. Dememew, D. Assefa, T. Gudina, A. Kassa, T. Letta, B. Ayele, Y. Tadesse, B. Tegegn, D. G. Datiko, C. Negeri, A. Bedru, E. Klinkenberg

**Affiliations:** 1 KNVC Tuberculosis Foundation/Management Sciences for Health USAID/Challenge TB Project, Addis Ababa, Ethiopia; 2 Federal Ministry of Health of Ethiopia, National TB Program and Child Health Unit, Addis Ababa, Ethiopia; 3 Addis Ababa City Administration Health Bureau, Addis Ababa, Ethiopia; 4 KNCV Tuberculosis Foundation, The Hague, Netherlands; 5 Department of Global Health and Amsterdam Institute for Global Health and Development, Location Academic Medical Center, Amsterdam University Medical Centers, Amsterdam, Netherlands; Rutgers University, UNITED STATES

## Abstract

**Background:**

Aligned with global childhood tuberculosis (TB) road map, Ethiopia developed its own in 2015. The key strategies outlined in the Ethiopian roadmap are incorporating TB screening in Integrated Maternal, Neonatal and Child Illnesses (IMNCI) clinic for children under five years (U5) and intensifying contact investigations at TB clinic. However, these strategies have never been evaluated.

**Objective:**

To evaluate the integration of tuberculosis (TB) screening and contact investigation into Integrated Maternal, Neonatal and Child Illnesses (IMNCI) and TB clinics in Addis Ababa, Ethiopia.

**Methods:**

The study used mixed methods with stepped-wedge design where 30 randomly selected health care facilities were randomized into three groups of 10 during August 2016-November 2017. The integration of TB screening into IMNCI clinic and contact investigation in TB clinic were introduced by a three-day childhood TB training for health providers. An in-depth interview was used to explore the challenges of the interventions and supplemented data on TB screening and contact investigation.

**Results:**

Overall, 180896 children attended 30 IMNCI clinics and145444 (80.4%) were screened for TB. A total of 688 (0.4%) children had presumptive TB and 47(0.03%) had TB. During the pre-intervention period, 51873 of the 85278 children (60.8%) were screened for TB as compared to 93570 of the 95618 children (97.9%) in the intervention (p<0.001). This had resulted in 149 (0.30%) and 539 (0.6%) presumptive TB cases in pre-intervention and intervention periods (p<0.001), respectively. Also, nine TB cases (6.0%) in pre-intervention and 38 (7.1%) after intervention were identified (p = 0.72). In TB clinics, 559 under-five (U5) contacts were identified and 419 (80.1%) were screened. In all, 51(9.1%) presumed TB cases and 12 (2.1%) active TB cases were identified from the traced contacts. TB screening was done for 182 of the 275 traced contacts (66.2%) before intervention and for 237 of the 284 of the traced (83.5%) under intervention (p<0.001). Isoniazid prevention therapy (IPT) was initiated for 69 of 163 eligible contacts (42.3%) before intervention and for 159 of 194 eligible children (82.0%) under intervention (p<0.001). Over 95% of health providers indicated that the integration of TB screening into IMNCI and contact investigation in TB clinic is acceptable and practical. Gastric aspiration to collect sputum using nasogastric tube was reported to be difficult.

**Conclusions:**

Integrating TB screening into IMNCI clinics and intensifying contact investigation in TB clinics is feasible improving TB screening, presumed TB cases, TB cases, contact screening and IPT coverage during the intervention period. Stool specimen could be non-invasive to address the challenge of sputum collection.

## Introduction

There were an estimated 1.1 million children with TB globally, and this is about 11% of the estimated 10 million total TB cases in 2018 [1]. TB is one of the top 10 contributors to U5 mortality in TB endemic countries such as Ethiopia [2]. Ethiopia is one of the 30 high TB burden countries with an estimated TB incidence of 151/100,000 population in 2018 [1]. Accoridng to unpublished national TB report during July 2018-June 2019, there were 10,080 children less than 15 years among the 100,782 (10.0%) new TB cases where 2712 were children U5, representing 2.7% of total new cases in Ethiopia [3].

According to the 2019 global TB report, a third of estimated TB cases in Ethiopia were not detected [1]. Of the missed TB cases, 11,918 (20.0%) were estimated to be among children [3].

TB diagnosis in children is more difficult than in adults [4–6] due to the non-specific presentation of TB in children in the form of pneumonia, wheeze, and fever of unknown origin. In many developing countries, there is limited capacity, especially at the lower levels of the health care system, to suspect and diagnose TB in children. Also, systematic investigation of child contacts of adult pulmonary TB cases, an entry point to identify tuberculosis disease in children, is occasionally practiced. This causes a huge missed opportunity [7, 8]. Younger children rarely produce adequate sputum samples, reducing the possibility of utilizing rapid and sensitive TB diagnostic tools like GeneXpert for children [9].

In high TB burden settings like Ethiopia, there are opportunities to identify children with presumptive TB in the framework of IMNCI clinics or integrated community case management (ICCM). Though these clinics prioritize the identification and management of acute childhood diseases; such as diarrhea, malnutrition, pneumonia and fever, TB is likely affecting a number of children evaluated in these clinics. Besides, TB could be masked by symptoms of these accute ilnessses [10]. Therefore, these clinics could harbour missed TB cases in children [11]. Yet an important step towards improving identification, prevention and management of TB in children is the provision of integrated care in IMNCI and ICCM platforms [12].

Accordingly, Ethiopia was the first African country to develop a national childhood TB roadmap following the global childhood TB road map in 2015 [13–15]. The key strategies outlined in the Ethiopian roadmap is integrating TB screening, diagnosis and prevention services into IMNCI clinics at the primary health care level and intensifying contact investigations at the TB clinics. These strategies can increase TB case detection in children and provide an opportunity to provide IPT [13, 15, 16]. The federal ministry of health of Ethiopia (FMOH) started implementing both strategies in Addis Ababa as a pilot before scaling up. This provided an opportunity to evaluate these interventions through implementation research.

## Materials and methods

### Settings

The study was carried out in Addis Ababa, the capital city of Ethiopia, with an estimated population of 3.9 million in 2017/2018 [17]. According to unpublished national TB report of 2018/2019, a total of 4,070 TB cases (116 /100, 000) were notified in Addis Ababa, of whom 191 (4.7%) were children less than 15 years of age [3].

Health centers are the first entry point to the formal health system, and they are the centerpiece of primary health care services in Ethiopia. One of their key roles is the provision of curative IMNCI services, such as treating children with diarrhea, pneumonia, and malnutrition. Cough triage, TB screening, diagnosis, and treatment are part of the TB service provided at the primary health care level with little focus on childhood TB.

### Study design

A mixed method study was adopted to evaluate the programmatic intervention outlined below. For the quantitative part, a stepped-wedge design was applied as this is an optimal design to evaluate phased in interventions [18]. In depth interview and field notes (qualitative data) supplemented the quantitative data collection. For the stepped wedge design, the units of comparison were groups of randomly assigned health facilities that moved over to the intervention together. Allocation was not concealed, but clinicians, and TB and IMNCI officers were blinded to the order of entry into the intervention till each group of health centers were enrolled to the interventions [19].

**Fig 1:**
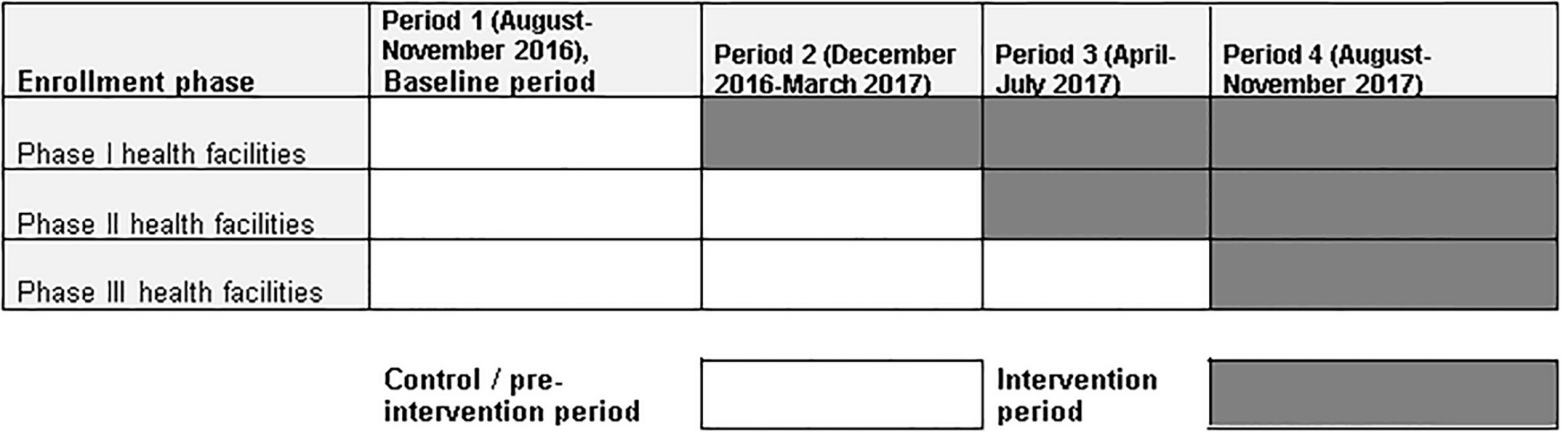
Schematic representation of the stepped wedge design of the implementation study in 30 health centers in Addis Ababa, August 2016-November 2017. Fig 1 shows the four periods, each with the length of four months. The first period (August- November 2016) was the baseline period where all health facilities served as control. December 2016-March 2017 was phase I where the first group of 10 health facilities (phase I health facilities) were enrolled into intervention. During this phase the rest of 20 health facilities served as control in the control period. April-July 2017 was a period when the second group of 10 health facilities (phase II) were enrolled into intervention. Here, phase I &II health facilities served as intervention health facilities in the intervention period, whereas the remaining 10 health fasciitis (phase III facilities) served as a control in the control period. Finally, in August-November 2017 phase III facilities entered the intervention.

The study was undertaken over a period of 16 months, and facilities transitioned in four- month intervals from pre-intervention to intervention period (Fig 1).

### Sampling

Initially, all of the 100 health centers in Addis Ababa were listed with their annual patient load in the IMNCI clinic. Fifty health centers that reported more than 500 U5 children per year were included in the sampling frame. From these, 30 were randomly selected as the study health facilities. Subsequently, the 30 facilities were randomly assigned to three groups, each with 10 health centers, which started the intervention phase by phase.

Health care workers from each group of 10 health centers were trained and made ready to start the intervention at four-month interval (Fig 1). Preparation for and initiation of the interventions were during the fourth month of the previous phase and the first month of the upcoming phase.

### Interventions

The interventions consisted of the integration of TB screening into IMNCI clinics, and enhanced childhood TB case finding in the TB DOT unit (TB DOTS) clinics through contact investigation. To guide the intervention, desk reference materials in the form of pediatric TB job aids, updated IMNCI registers with a TB screening column and registers for contact investigation and IPT provision were developed and supplied. Sensitization on the implementation study was conducted for child-health and TB program officers and heads of the study health facilities. This was followed by a three-day basic childhood TB training for health care providers from the IMNCI and TB DOTS clinics to introduce the intervention. Nasogastric (NG) tubes were provided to each study health facility. Research physicians demonstrated and mentored Health care workers (HCWs) on how to perform the nasogastric aspiration (NGA) procedure. Onsite coaching of health providers followed classroom demonstration till the HCWs confidently carried out the procedure. The research team also monitored the performance of each study health facility and record keeping practice monthly.

TB screening and diagnosis, and contact tracing were based on the existing national guidelines and algorithms [20]. A child was identified as a presumptive TB case if cough, fever, failure to gain weight or contact history was reported. As per national policy, all children with presumed TB cases received GeneXpert MTB/Rif (GXP) as a primary diagnostic test. If the GXP was negative, contact history with an infectious pulmonary TB (PTB) case, suggestive chest X-ray and the presence of clinical signs of TB were used as criteria to diagnose TB in a child. The presence of at least two of these made a clinical diagnosis of TB in children. Those children identified to have active TB cases were started on TB treatment at TB DOTS clinic. To enhance contact tracing, all U5 contacts of PTB index cases identified at the TB DOTS clinics underwent evaluation for TB as outlined above. As part of contract tracing, children with screen negative or non-presumed TB case, thus without suggestion of TB, were eligible for prophylaxis and offered IPT (S1 Fig).

### Data collection

Baseline data collection started in all study sites to document the practice of TB screening over a four-month period before the first group of 10 health facilities entered to the intervention. This baseline assessment was carried out using key outcome indicators, such as the number of presumed TB cases identified, the number of NGA undertaken, the number of TB cases identified at IMNCI clinics, the number of eligible contacts traced and screened, and the number of eligible U5 contacts started on IPT. During the project implementation, the researchers provided a standardized set of tools to capture the childhood TB data. From the study health facilities, HCWs were recruited and trained as data collectors. At the end of every fourth month, the trained HCWs collected data from the records of the study health facilities.

In addition, in-depth interviews (IDIs) were performed with 30 HCWs and 11 heads of study health facilities. Those on duty on the interview day were selected. The study coordinator conducted the IDIs until saturation, i.e. until no new information or views were obtained from subsequent interviews.

The IDIs were conducted twice during the project period, at the beginning (August-November 2016) and at the end (August-November 2017). IDIs were guided by a developed interview guide inquiring about the advantages and barriers of integration of TB screening into IMNCI and contact investigation to TB clinics. The study coordinators kept field notes of the observation during the data collection. The IDI and field notes were captured in English, though the interview was conducted in the local language, Amharic, for convenience.

At the end of the study, assessment on the feasibility of integration of TB screening into IMNCI and intensified contact investigation in TB clinics was done through a structured questionnaire among the selected set of 190 parents/caretakers, 80 health care providers and 30 heads of the study health facility. The data collectors interviewed the parents and caretakers when they showed up at IMNCI and TB clinics with their children, and the HCWs while on duty.

### Data quality

Data collectors attended a one day training on how to extract data using the checklist. The researchers checked data consistency everymonth during mentoring visits and every quarter during supportive supervision to the study sites. The supervision was undertaken jointly with the staff from KNCV Tuberculosis Foundaiton, the national TB program (NTP) and the Maternal and Child Health Unit of FMOH and Addis Ababa regional Health bureau (AARHB). Double data entry was done for all extracted data. The quality control of this process was performed by the study coordinator. Before data analysis was commenced, data cleaning and validation of the entered data was done by checking for data completeness, presence of outliers and inconsistencies. The summary of findings from IDIs, and field notes of observation at the health facilities were re-checked with IDI participants.

### Data analysis

Data entry for the quantitative part was done using EPI info (Version 7.2.2.16; Atlanta, Georgia: Centers for Disease Control and Prevention; 2018). Data analysis was done using STATA (Version 13; College Station, Texas: Stata Corp; 2013).

The main indicators such as the proportion of children screened for TB from patients at IMNCI clinics, the proportion of presumed TB cases and TB cases identified from the screened, the proportion of presumptive TB cases with NGA procedure done and the coverage of IPT (number of children started on IPT/number of children eligible for IPT) were compared using the two sample proportion test over the 16 months control and 16 months intervention period. The mean number of children screened, identified with presumptive TB and detected to have active TB during the control (pre-intervention) period and the intervention period per the study site was also compared using two samples mean comparison test or t–test. The 95% CI and p-value (less than 0.05) were used to assess statistical significance.

The manual theme-based word data analysis was done based on the IDIs and field notes. Subsequently, the quantitative and qualitative findings were triangulated. Eventually, the feasibility (acceptability and practicality) of the integration of childhood TB screening to U5 clinics was described quantitively using frequency and proportion.

### Ethical considerations

Ethical clearance was obtained from the Ethical Review Board of Addis Ababa City Health Bureau (AACHB). Support letters from AACHB to the sub-cities health bureaus, and then from sub-cities to the study health facilities were written to obtain permission to conduct the study and gain access to the childhood TB data of each the study participants. After the heads of the study facilities were briefed on the aim and methodology of the study, they provided a permission to include their health facility in the study. As this was a routine strategy being evaluated, no written consent was obtained for each child to take part in the study. The ethics committee waived the requirement for informed consent, and the data of each child was anonymized as well. Children diagnosed with TB were provided with TB treatment, and children eligible for preventive therapy were provided with IPT per the national guidelines.

## Results

Over the 16 months period, a total of 181,455 children attended the 30 health facilities. Of these, 145,862 (80.4%) were screened for TB where 739 (0.4%) were presumed TB cases and 59 (0.03%) were TB cases. Over the study period, 125 successful NGA procedures were performed, and 12 (9.6%) of these were confirmed to be MTB by GXP. That is, only 12 of the 59 TB cases (20%) were bacteriologically confirmed.

A total of 559 U5 contacts were traced from the 1603 index cases, an average of one contact per three index cases. From the traced contacts, 419 (80.1%) were screened for TB. Three hundred fifty-seven were eligible for IPT, of whom 228 (63.9%) were started on IPT (Table 1).

**Table 1:** TB screening, identification of presumptive TB and TB cases, and contact investigation and TB preventive therapy efforts in the 30 study facilities in Addis Ababa Ethiopia, August 2016- November 2017.

Ser No.	Variables	Overall frequency (proportion)	Control period Frequency (proportion)	Intervention period Frequency (proportion)	Two-sample test of proportion, p-value (intervention vs control period)
1	**Overall (IMNCI unit and TB DOTS)**				
1.1	Children in attendance	181,455	85553	95902	
1.2	Children screened for TB (proportion screened)	145,862 (80.4%)	52055 (60.80%)	93807 (97.90%)	<0.001
1.3	Presumptive cases identified (proportion from screened)	739 (0.5%0	154 (0.3%)	585 (0.6%)	<0.001
1.4	NGA procedure carried out (proportion of those with presumptive TB)	125 (1.0%)	18 (11.7%)	107(18.1%)	0.06
1.5	TB cases detected (proportion from presumed TB cases)	59 (8.0%)	11 (7.1%)	48 (8.2%)	0.66
2	**IMNCI unit**				
2.1	children participated	180,896	85278	95618	
2.2	children screened for TB (proportion screened)	145443 (80.4%)	51873 (60.8%)	93570 (97.9%)	<0.001
2.3	presumptive cases identified (proportion from screened)	688 (0.5%)	149 (0.3%)	539 (0.6%)	<0.001
2.4	NGA procedure carried out (proportion of those with presumptive TB)	105 (15.3%)	18 (12.1%)	87 (16.1%)	0.22
2.5	TB cases detected (proportion from presumed TB cases)	47 (6.8%)	9 (6.0%)	38 (7.1%)	0.67
3	**TB DOTS**				
3.1	Number of index cases	1,603	684	919	
3.2	U5 contact children traced	559	275	284	
3.3	contacts screened (proportion screened)	419 (75.0%)	182 (66.2%)	237 (83.5%)	<0.001
3.4	Presumptive TB cases identified (proportion from screened)	51 (12.2%)	5 (2.7%)	46 (19.4%)	<0.001
3.5	NGA procedure done (proportion of those with presumptive TB)	20 (39.2%)	0 (0%)	20 (43.5%)	NA
3.6	TB cases detected (proportion from presumed TB cases)	12 (23.5%)	2 (40.0%)	10 (21.7%)	0.36
3.7	Eligible U5 children eligible for IPT	357	163	194	NA
3.8	Children started on IPT (proportion from eligible)	228 (63.9%)	69 (42.3%)	159 (82.0%)	<0.001

### IMNCI clinic

**Fig 2:**
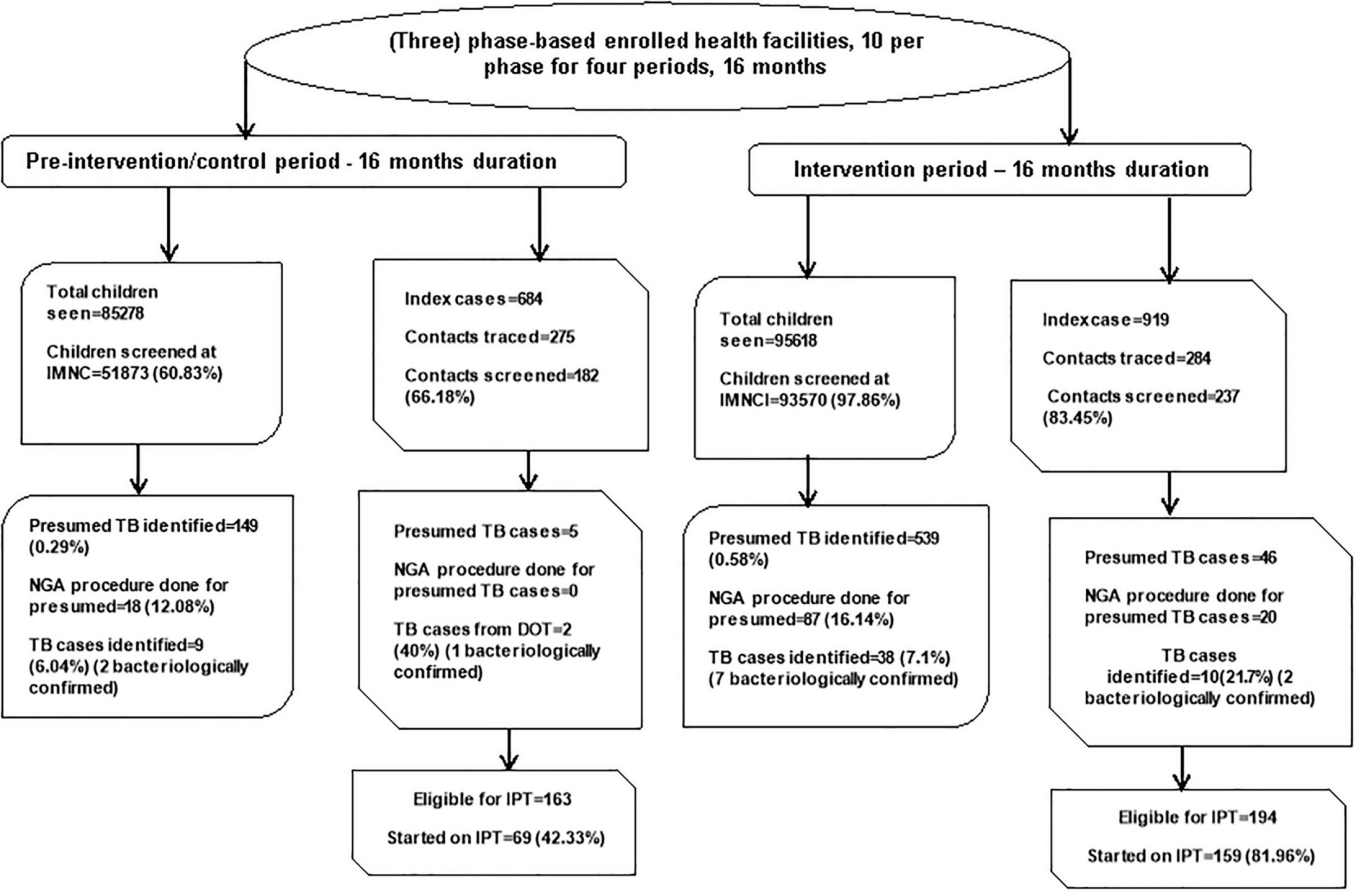
Summary of the findings: TB screening, evaluation, and diagnosis at IMNCI; and contact tracing, screening, and initiation of TB preventive therapy at TB DOTS in Addis Abba Ethiopia, August 2016-November 2017. Fig 2 shows the flow of TB screening and contact investigation activities based on the control and intervention period. For each period, there was TB activities at IMNCI and TB DOTs clinics. At IMNCI clinic, TB screening, identified presumed TB cases and TB cases were reported. At TB DOTS, number of index cases, contacts traced and screened, and IPT coverage were reported.

Of 180,896 children seen at IMNCI, 85,278 (47.1%) visited the study health facility during the pre-intervention period while 95,618 (52.9%) of them were seen during the intervention. A total of 145,443 (80.4%) of these children were screened for TB. TB screening was undertaken for 51,873 (60.8%) of children during the pre- intervention period and for 93,570 (97.9%) of the children under the intervention (p<0.001). From the overall screened children at IMNCI clinics, 688 (0.5%) children had presumptive TB cases where149 presumptive TB cases (0.3%) were identified among children screened duringthe control period while 539 (0.6%) were detected among children screened during the intervention (p<0.001). NGA was performed in 105 (15.3%) of the presumed TB cases. The procedure was carried out for 18 (12.1%) of the presumed TB cases identified during the pre- intervention and, it was done for 87 (16.1%) of the presumed TB cases during the intervention period (p = 0.22). A total of 47 TB cases were identified from the 688 presumed TB cases (6.8%). Nine (6.0%) were from the presumed TB cases detected before the intervention while 38 (7.1%) were from the presumed children detected under the intervention period (p = 0.67) (Table 1 and Fig 2).

### TB DOTS clinic

Of the 1603 index cases at TB DOTS clinics, 684 (42.7%) were identified in the pre-intervention and 919 (53.3%) during the intervention period (p<0.001). TB screening was done for 182 of the 275 traced contacts (66.2%) before the intervention and for 237of the 284 traced (83.5%) after the intervention (p<0.001). Overall, 51 of the screened contacts in TB DOT (12.2%) had presumed TB cases, five (2.7%) among the screened before the intervention and 46 (19.4%) after the intervention (p<0.001). Before the intervention, no NGA procedures were undertaken in the TB DOTS clinic while 20 NGA procedures were performed during the intervention period. A total of 12 TB cases, 23.5% among the presumed TB cases, were identified in the TB DOTS clinic during the study period, two (40%) were among the presumptive TB cases before the intervention and 10 (21.7%) under the intervention (p = 0.36). IPT was initiated for 69 (42.3%) of eligible U5 children in the control period as compared to 159 (82.0%) during the intervention (p<0.001) (Table 1 and Fig 2). The details of TB activities at IMNCI and TB DOTS clinics in the 30 health facilities are described in S1 Table.

Table 2 shows the difference in the mean of children screened, presumed TB case identified, NGA procedure done, contacts traced, contacts screened and put on IPT in each study health facility during the pre-intervention and intervention periods. During the intervention period an average of 697 more children in each study health facility were screened (p<0.001). An average of eight more children with presumptive TB were identified (p<0.001), two more NGA procedures were done (p<0.001) and 01 more TB cases were identified (p<0.001) during the intervention period as compared to the pre-intervention in each study health facility. At IMNCI clinics, the mean difference in the number of children screened for TB, identified presumptive TB cases, performed number of NGA procedures and notified TB cases during the intervention period, as compared to the pre-intervention period, was statistically significant (p<0.001). In addition, the proportion of U5 children screened for TB was increased by 47.2% (95% CI 39.5%-54.9%) after intervention, by 47.3% (95% CI 39.6–55.0%) at the IMNCI clinics and by 27.9% (95% CI18.7–37.0%) at TB-DOTS. Also, IPT coverage was increased by 42.2% (95% CI 27.8–56.6%) after the intervention (Table 2). Further study health facility based detail of TB activities at IMNCI and TB DOTS based on the control and intervention periods is shown in S2 Table.

**Table 2:** Comparison of TB activities during the pre-intervention/control and intervention period per the study facility in Addis Ababa, Ethiopia, August 2016- November 2017.

Ser No.	Variables	Mean Difference per study health facility (after and before intervention)	95% Confidence Interval of mean difference		t-value	Sig. (2-tailed)
			Lower	Upper		
1	**Overall comparison**					
1.1	Total children involved	172	-128	471.7	1.1	0.26
1.2	Total screened	697.9	400.6	991.1	4.7	<0.001
1.3	Total presumptive TB case	7.6	5.1	10.03	6.04	<0.001
1.4	Total NGA procedure done	1.5	0.7	2.3	3.5	<0.001
1.5	Total TB cases identified	0.6	0.3	0.9	4.3	<0.001
1.6	% screened	47.2	39.5	54.9	12.1	<0.001
2	**IMNCI unit**					
2.1	No of U5 (IMNCI) children involved	172.3	-127.1	471.7	1.1	0.26
2.2	% screened at _IMNCI	47.3	39.6	55	12.1	<0.001
2.3	NGA done at IMNCI	1.2	0.4	1.9	2.9	<0.001
2.4	TB cases at IMNCI	0.5	0.2	0.7	4	<0.001
3	**TB DOTS unit (Contact investigation and preventive therapy)**					
3.1	% Contact screened	27.9	18.7	37	6.03	<0.001
3.2	%IPT coverage	42.2	27.8	56.6	5.8	<0.001

### Challenges of childhood TB integration into under-five clinics

Health care providers usually refer children with presumptive TB to higher level health facilities. Because of the shortage of skilled manpower to diagnose TB in children, 22 of the 30 health facilities (73.0%) indicated to send children to higher level hospitals for evaluation and confirmation of TB during the baseline assessment. In addition to the non-specific presentation of TB in a child, this referral is mainly due to the lack of skilled HCWs to carry out the procedure of NGA. This was justified as,

*“The failure to get a child with textbook symptoms and signs of TB*… *and few staff with the skill of conducting nasogastric aspiration could be ascribed to the difficulty of identifying TB among the children”*

*M*, *38*, *Head of a health center*.

The turnover of trained HCW in the IMNCI clinics was also pointed out as one of the challenges. In addition, HCWs reported to be overstretched due to the large number of U5 visits compromising quality of care and attention for TB screening. One of the participants stated,

*‘‘*… *HCWs could be busy dealing with many U5 visits* … *There is a limited training on childhood TB for health care workers on the newly updated childhood TB diagnostic algorisms*.*”*

*Female (F)*, *30*, *head of a health center*.

Irrespective of the intervention, the health care providers indicated to have a low index of suspicion for presumptive TB cases of childhood TB. This was thought partly because a chronic cough was rarely identified in children through regular follow-up. Though the IMNCI guidelines indicate that parents should come back for follow up if the cough does not improve within two weeks, this often does not happen. Parents with children having respiratory tract infections usually seek for better care at private clinics or higher-level facilities, making it impossible to strict follow-up of pneumonia, malnutrition or URTI at health centers as per the IMNCI guidelines. The head of one of the study health facility described this as follows,

*“If a child does not improve with common treatment as recommended by IMNCI*, *s/he is likely to be taken to private clinics* …. *and may not return to the IMNCI clinic*… *Families prefer to take the child (that does not improve) to hospital if the child has a cough or other signs and symptoms indicative of the presumed TB case*; *hence*, *difficult to observe chronic cough in kids”*

*M*, *40*, *head of health center*.

Therefore, in most cases, one cannot retain children long enough for further investigations or for subsequent screening to identify presumptive TB cases.

### Feasibility of integrating childhood TB into IMNCI

#### Acceptability

At the study health facilities, more than 95.0% of the parents/guardians, health care providers and heads of the health facilities indicated they were comfortable with an integrated service delivery of TB screening and evaluation at IMNCI clinics and contact investigation at TB DOTS clinics. More than 94.0% of the clients and HCWs, and all facility heads said that they had a positive perception of the integration and none of them had negative feeling related to the integration of TB screening.

#### Practicality

Both the health care providers (95.0%) and the heads of the health facilities (100.0%) indicated that the implementation of TB symptom screening and contact investigation at IMNCI and TB DOTS clinics was easy to put in practice. (Table 3).

**Table 3:** The assessment of feasibility of integrating TB screening and contact investigation activities into IMNCI and TB DOTS clinics, August 2016- November 2017 Addis Ababa.

Ser No.	Feasibility	Clients who responded yes (N = 190)	Service providers who responded yes (N = 80)	Heads of the study health facilities who responded yes, (N = 30)
**1**	**Acceptability (Y/N)**			
1.1	Satisfied with availability of TB screening and contact investigation services at the same place as IMNCI and TB DOTs services	95.0	95.7	95.2
1.2	Is it appropriate to integrate TB screening and contact investigation services with IMNCI and TB DOTs services	94.7	90.9	100.0
1.3	Perceived positive effects of integrated TB screening and contact investigation services on IMNCI and TB DOTs services	94.4	95.2	100.0
**2**	**Practicality (Y/N)**			
2.1	Is it practical to implement TB symptom screening, clinical evaluation, and treatment for TB	NA	95.2	100
2.2	Are the suggested process, tools, and SOPs for TB management in IMNCI and TB DOTs setting easy to adopt	NA	94.7	100
2.3	Is delivery of integrated TB services through IMNCI and TB DOTs sustainable considering cost and human resources?	NA	86.4	89
2.4	Do integrated TB screening and contact investigation services disrupt implementation of routine IMNCI and TB DOTs services?	NA	14.3	17

## Discussion

We showed that the integration of TB screening into IMNCI and enhanced contact investigation at TB DOTS clinics is feasible, resulting in improved TB screening from 60.8% to 97.9%, identification of presumed TB cases from 0.3% to 0.6% and TB case detection from 7.1% to 8.2%. An additional 37 children with TB were diagnosed in the 30 clinics as part of the 16-month intervention. Enhancing contact investigation and improving the link between the TB DOTS clinic and the IMNCI clinic brought about 90 additional children accessing IPT and an additional 8 TB cases being detected over the study period reducing the potential of missed TB cases. In addition, the proportion of contacts screened was improved from 66.2% to 83.5%, while the coverage of IPT was increased from 42.3% to 82.0%. Nevertheless, challenges were also reported; the key ones being frequent turnover of trained staff and difficulty of obtaining sputum samples from young children with presumptive TB. High staff turnover is a common problem in developing country settings [21] and should be addressed as part of the further roll out of this intervention and in the general health care system. The difficulty of obtaining sputum samples has been previously reported [6] and is known for U5 children. At present, stool, which is easier to obtain and not invasive is being considered as an alternative sample for diagnosis of TB in children [22, 23] and investigated in a follow up project [24] in Ethiopia.

At the end of the study period, TB screening was conducted for over 98% of all children that attended the IMNCI clinics. This is much higher than findings from Uganda, where 47.0% of those <15 years were screened for TB symptoms [25]. In the same Ugandan study, however, 15.0% of the screened children were identified as presumptive TB, which is much higher than the 0.6% in our study. The lower screening coverage in Uganda may have resulted in a more targeted group of presumptive cases partly explaining the much higher proportion of presumed TB cases observed, as the study included many self-reported presumed TB cases. In our study, the proportion of TB screening and identified presumed TB cases could have been improved further had it been not for the high workload and high turnover of trained staff. Addressing such challenges could potentially further enhance the diagnosis of TB in children. Hence, on-the-job orientation on the importance of universal screening and evaluation of sick children for TB should become a routine activity.

It was shown that about four times less presumed TB cases were identified in the pre-intervention period, suggesting opportunities are currently being missed in routine childhood TB activities. This is also reflected in a community-based study in Ethiopia which showed that under routine care, opportunities are being missed to diagnose TB. This is because all presumed TB cases are not being identified and referred to a corresponding health center by health extension professionals (HEPs) leading to underdiagnosis of TB cases [26, 27]. According to a study by Tulloch and colleagues in Ethiopia, presumed TB cases often are not evaluated for active TB diseases even though they have a chronic cough. This is contributing to the missed TB cases [28]. Identification of presumptive TB in children largely depends on contact history with infectious TB cases, recurrent and repeated pneumonia, and failure to respond to the standard therapeutic therapy [29–31]. Those in contact with undiagnosed chronic coughers in the neighborhood or school should be considered to find all TB cases in children. Also, it is essential to avoid pre-diagnostic lost to follow up among children potentially showing signs and symptoms of TB [32]. That is, if all presumed TB cases that are treated for other non-TB cases but are not responding to the treatment and become deteriorated, these could systematically be followed by the initial health facility. Doing so, children with presumptive TB could more easily be identified and subsequently be evaluated for TB.

Twenty-nine additional TB cases were identified in the intervention period at the IMNCI units, showing the potential of missed childhood TB cases under current routine conditions. Similarly, a study in Pakistan found that systematic verbal screening combined with contact tracing with appropriate management services resulted in a three-fold increase in pediatric TB case notification, cases that might have otherwise been missed [33]. In our study, it is important to note that only 88 NGA samples were collected out of the 454 presumed TB cases that were eligible for NGA procedure. The lower NGA performance was mainly due to lack of skill in the health care workers. Yet, if specimen collection and handling protocols could be strictly adhered to, NGA GXP can be highly specific for diagnosis of PTB in children [34] and we could have identified more TB cases.

Our study indicated that 10% of the NGA test results were MTB detected, resulting in a bacteriologically confirmed diagnosis. This is similar to a study in Botswana whereby 121/1274 NG samples (9.5%) were confirmed by culture [35]. Other studies found lower levels of bacteriological confirmation; for example, a study in India found 5.8% [36]. The difference could be due to the specific settings, skill differences or characteristics of the study population.

Our study also showed that enforcing contact investigation in the TB DOTS clinic can serve to increase TB case detection and coverage of preventive therapy. There were an additional 17.3% of child contacts screened, 39.6% more children started on IPT and 8 more TB cases identified. Similarly, a facility-based study on childhood TB screening in rural Pakistan identified 390 additional children with TB through contact tracing [33]. Studies from the southern region of Ethiopia [37], Amhara and Oromia regions [38–40] have shown that contact tracing can serve as a good entry point for IPT provision. However, this has not been replicated in all countries as shown by a study in Botswana attributing the low coverage of IPT to a poor implementation of contact tracing [41]. A study in Tanzania, conducted a year after a training on childhood TB, indicated that three times as many trained as untrained health care providers reported having ever prescribed IPT to a child [42]. Hence, consistent and continuous capacity building of health care workers at primary health care facilities is needed to improve and sustain contact investigation and provision of IPT for TB. The need to prepare a standard counseling tool that could assist in explaining parents the benefits of contact tracing and IPT at TB DOTS clinic is suggested.

We also indicated that improving childhood TB screening and diagnosis is feasible and practical after an intense training and creation of awareness on childhood TB among health care providers at the primary health care units, and this is similar to other studies [42, 43]. However, the required capacity building should not be limited to the health facilities, but needs to also involve community health care workers [44] to facilitate the integration of childhood TB into the ICCM platform. The integration of childhood TB into the child health platform was shown to be acceptable retaining more children to care in Malawi [45]. In Pakistan, the introduction of the intensified childhood TB care into the national TB program policy improved TB case finding as well as treatment outcomes [46]. Also, our study demonstrated that trained and mentored clinicians at child health clinics could screen, confirm TB diagnosis and link to treatment, yet the extent of the practical integration at primary care units should be explored further. Besides, the facility and community level routine monitoring of the childhood TB integration into sick child platforms necessitates the coordination and collaboration work with primary health care unit director, HEWs and other community health workers.

Our study is one of a few studies evaluating the implementation of the integration of childhood TB screening into IMNCI clinics and contact investigation into TB DOTS units using a mixed method study. However, the findings should be interpreted cautiously because the study did not evaluate the integration of TB screening and contact investigation at the other childhood clinics. Therefore, the positive results achieved with the current study could be further expanded by involving other childhood health services such as nutrition, vaccination or, pediatric inpatient units as well as at the community level.

## Conclusions

This demonstration work suggested that the implementation of integration of childhood TB screening to IMNCI and intensified contact investigation at TB DOTS clinics could improve TB diagnosis, and is acceptable and practical in Ethiopia. We have shown that appropriately trained frontline health workers at primary heath care unit level can significantly improve childhood TB diagnosis. Collecting sputum samples to confirm TB in children is difficult and alternative samples such as stool, which are easier to collect without invasive procedures, could be considered. Finally, contact investigation should be enhanced as an entry point for increasing IPT coverage for U5 children. In addition to the IMNCI, further integration of childhood TB screening into other childhood clinics such as nutrition therapy, expanded program on immunization (EPI), maternal and child health (MCH), pediatric wards or in patients, and pediatric emergency department clinics in health facilities could be considered to increase the coverage of screening U5 children.

## Supporting information

S1 Fig The flow chart and the algorism of TB screening, TB diagnosis, contact tracing and screening in Addis Ababa health facilities, August 2016- November 2017. S1 Fig shows that presumed TB cases in children could show up at IMNCI/U5 or TB DOT clinic. In both clinics, history of contact to infectious TB cases (or PTB) is used to identify children exposed to TB infection. PTB are smear negative and smear positive TB cases around which contacts are traced to be screened for TB. Eligible U5 children for IPT service were those with non-presumed TB cases and those with no contraindication to INH.
(TIF)

S1 Table Phase and period-based activities at IMNCI and TB DOTS clinics in the 30 health facilities, August 2016-November 2017, Addis Ababa. The table indicates the three phase health facilities (Phase I, II &III) enrolled to the study/intervention. Phase I means the first 10 health facilities that were enrolled to intervention during December 2016-March 2017. Phase II means the second batch of 10 health facilities enrolled to intervention and it was from April-July 2017. Phase III is the period when the last 10 of the health facilities got into the intervention and it is August- November 2017. Note that the period of August-November 2016 was where all health facilities served as a control or baseline. The darker box is for TB activities for the intervention period and the normal or white sections is data for the control/baseline period.
(DOCX)

S2 Table Overall and average TB activities at IMNCI and TB DOT based on the control and intervention periods per each four months per study health facilities of Addis Ababa, Ethiopia, August 2016-November 2017. This table indicates the total/sum, the ranges, and the means with 95% CI of TB screening and contact investigation activities for the control and intervention period health facilities. The range and the mean are shown per the study health facilities over the four months period.
(DOCX)
