# Supplementary figures and images for: Evaluating the integration of tuberculosis screening and contact investigation in tuberculosis clinics in Ethiopia: A mixed method study
Source: PLoS One. 2020 Nov 19;15(11):e0241977. doi: 10.1371/journal.pone.0241977 (PMC7676707; doi:10.1371/journal.pone.0241977)

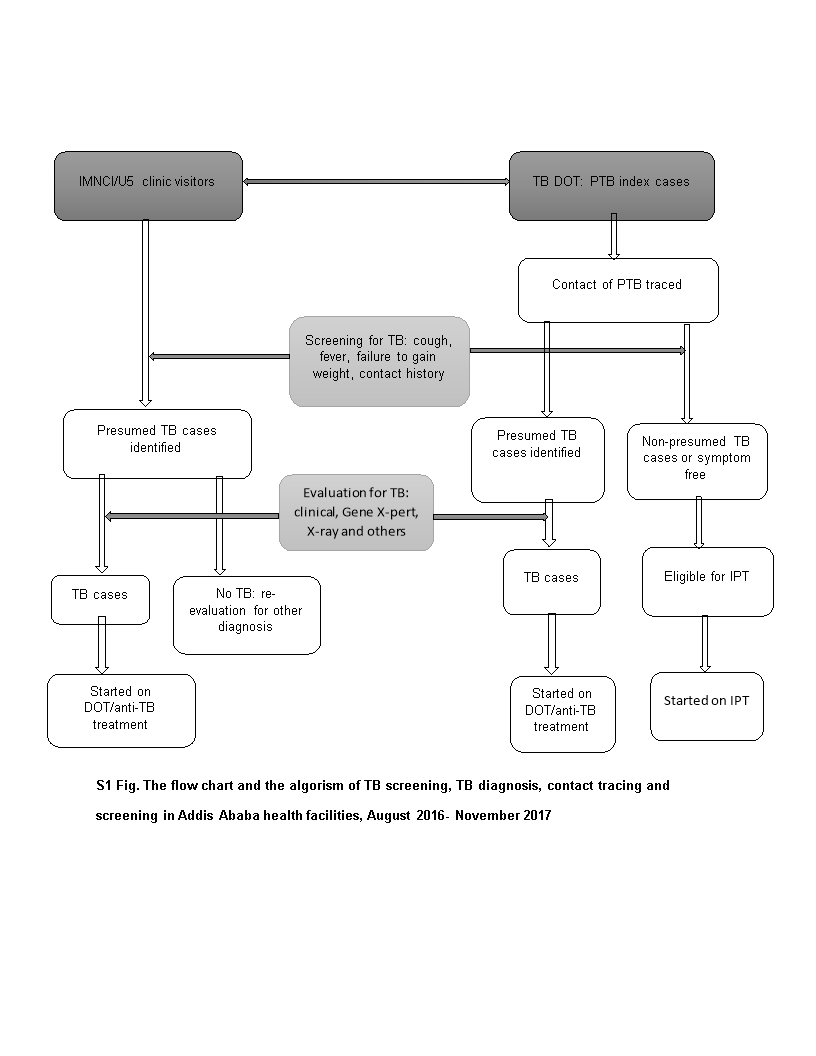

Supplement: S1 Fig — shows that presumed TB cases in children could show up at IMNCI/U5 or TB DOT clinic. In both clinics, history of contact to infectious TB cases (or PTB) is used to identify children exposed to TB infection. PTB are smear negative and smear positive TB cases around which contacts are traced to be screened for TB. Eligible U5 children for IPT service were those with non-presumed TB cases and those with no contraindication to INH. (TIF) [file pone.0241977.s001.tif]
