# Supplementary material for: Evaluating the integration of tuberculosis screening and contact investigation in tuberculosis clinics in Ethiopia: A mixed method study
Source: PLoS One. 2020 Nov 19;15(11):e0241977. doi: 10.1371/journal.pone.0241977 (PMC7676707; doi:10.1371/journal.pone.0241977)
Supplement: S1 Table — The table indicates the three phase health facilities (Phase I, II &III) enrolled to the study/intervention. Phase I means the first 10 health facilities that were enrolled to intervention during December 2016-March 2017. Phase II means the second batch of 10 health facilities enrolled to intervention and it was from April-July 2017. Phase III is the period when the last 10 of the health facilities got into the intervention and it is August- November 2017. Note that the period of August-November 2016 was where all health facilities served as a control or baseline. The darker box is for TB activities for the intervention period and the normal or white sections is data for the control/baseline period. (DOCX) [file pone.0241977.s002.docx]

**S1 Table. Phase and period based activities at IMNCI and TB DOT clinics in the 30 health facilities, August 2016-November 2017, Addis Ababa**

|  |  | **Pre-intervention or Control period** |  | **Intervention period** |  |  |  |
| --- | --- | --- | --- | --- | --- | --- | --- |
|  |  |  |  |  |  |  |  |
| **Variable** | **Enrollment phase** | **August-November 2016** | **December 2016_March 2017** | **April-July 2017** | **August-November 2017** | **Overall** | |
|  |  |  |  |  |  | **Control** | **Intervention** |
| **Total U5 children involved (IMNCI & U5 contact traced/TB DOTs)** | Phase I health facilities (HFs) | 29487 | 18974 | 18348 | 12355 | 85553 | 95902 |
|  | Phase II HFs | 10659 | 18368 | 17412 | 13424 |  |  |
|  | Phase III HFs | 9013 | 9013 | 9013 | 15389 |  |  |
| **U5 children IMNCI** | Phase I HFs | 29440 | 18917 | 18311 | 12322 | 85278 | 95618 |
|  | Phase II HFs | 10614 | 18299 | 17354 | 13388 |  |  |
|  | Phase III HFs | 8975 | 8975 | 8975 | 15326 |  |  |
| **U5F contacts traced** | Phase I HFs | 47 | 57 | 37 | 33 | 275 | 284 |
|  | Phase II HFs | 45 | 69 | 58 | 36 |  |  |
|  | Phase III HFs | 38 | 38 | 38 | 63 |  |  |
| **Total screened U5 (IMNC & TB DOT contacts)** | Phase I HFs | 19405 | 18608 | 17702 | 12355 | 52055 | 93807 |
|  | Phase II HFs | 5287 | 17998 | 17328 | 13389 |  |  |
|  | Phase III HFs | 2246 | 2246 | 4873 | 14425 |  |  |
| **U5 children_ screened _IMNCI** | Phase I HFs | 19371 | 18553 | 17667 | 12325 | 51873 | 93570 |
|  | Phase II HFs | 5266 | 17936 | 17274 | 13353 |  |  |
|  | Phase III HFs | 2227 | 2227 | 4846 | 14398 |  |  |
| **Contacts screened** | Phase I HFs | 34 | 55 | 35 | 30 | 182 | 237 |
|  | Phase II HFs | 21 | 62 | 54 | 36 |  |  |
|  | Phase III HFs | 18 | 19 | 28 | 27 |  |  |
| **Total presumptive TB cases** | Phase I HFs | 51 | 93 | 103 | 125 | 154 | 585 |
|  | Phase II HFs | 19 | 17 | 31 | 102 |  |  |
|  | Phase III HFs | 0 | 0 | 67 | 131 |  |  |
| **Presumptive at IMNCI** | Phase I HFs | 48 | 85 | 94 | 116 | 149 | 539 |
|  | Phase II HFs | 17 | 17 | 28 | 93 |  |  |
|  | Phase III HFs | 0 | 0 | 67 | 123 |  |  |
| **presumptive TB at DOT** | Phase I HFs | 3 | 8 | 9 | 8 | 5 | 46 |
|  | Phase II HFs | 2 | 0 | 3 | 8 |  |  |
|  | Phase III HFs | 0 | 0 | 0 | 10 |  |  |
| **Total NGA procedure** | Phase IHFs | 11 | 34 | 21 | 15 | 18 | 107 |
|  | Phase II HFs | 5 | 2 | 6 | 17 |  |  |
|  | Phase III HFs | 0 | 0 | 0 | 14 |  |  |
| **NGA procedure at IMNCI** | Phase I HFs | 11 | 30 | 17 | 13 | 18 | 87 |
|  | Phase II HFs | 5 | 2 | 4 | 11 |  |  |
|  | Phase III HFs | 0 | 0 | 0 | 12 |  |  |
| **NGA procedure at DOT** | Phase I HFs | 0 | 4 | 4 | 2 | 0 | 20 |
|  | Phase II HFs | 0 | 0 | 2 | 5 |  |  |
|  | Phase III HFs | 0 | 0 | 0 | 3 |  |  |
| **Total TB cases** | Phase I HFs | 3 | 8 | 14 | 8 | 11 | 48 |
|  | Phase II HFs | 2 | 5 | 4 | 7 |  |  |
|  | Phase III HFs | 0 | 0 | 1 | 7 |  |  |
| **TB cases at IMNCI** | Phase I HFs | 3 | 6 | 12 | 5 | 9 | 38 |
|  | Phase II HFs | 1 | 5 | 4 | 5 |  |  |
|  | Phase III HFs | 0 | 0 | 0 | 6 |  |  |
| **TB cases at DOT** | Phase I HFs | 0 | 2 | 2 | 3 | 2 | 10 |
|  | Phase II HFs | 1 | 0 | 0 | 2 |  |  |
|  | Phase III HFs | 0 | 0 | 1 | 1 |  |  |
| **Index cases** | Phase I HFs | 150 | 174 | 166 | 119 | 684 | 919 |
|  | Phase II HFs | 111 | 156 | 193 | 126 |  |  |
|  | Phase III HFs | 89 | 89 | 89 | 141 |  |  |
| **Contact screened** | Phase I HFs | 34 | 55 | 35 | 30 | 182 | 237 |
|  | Phase II HFs | 21 | 62 | 54 | 36 |  |  |
|  | Phase III HFs | 18 | 19 | 28 | 27 |  |  |
| **Eligible for IPT** | Phase I HFs | 25 | 47 | 27 | 22 | 163 | 194 |
|  | Phase II HFs | 19 | 62 | 51 | 28 |  |  |
|  | Phase III HFs | 19 | 19 | 19 | 19 |  |  |
| **Started on IPT** | Phase I HFs | 11 | 26 | 24 | 20 | 69 | 159 |
|  | Phase II HFs | 6 | 38 | 45 | 25 |  |  |
|  | Phase III HFs | 0 | 0 | 14 | 19 |  |  |
