# Supplementary material for: Evaluating the integration of tuberculosis screening and contact investigation in tuberculosis clinics in Ethiopia: A mixed method study
Source: PLoS One. 2020 Nov 19;15(11):e0241977. doi: 10.1371/journal.pone.0241977 (PMC7676707; doi:10.1371/journal.pone.0241977)
Supplement: S2 Table — This table indicates the total/sum, the ranges, and the means with 95% CI of TB screening and contact investigation activities for the control and intervention period health facilities. The range and the mean are shown per the study health facilities over the four months period. (DOCX) [file pone.0241977.s003.docx]

**S2 Table: The overall TB activities during the study period and mean of TB activities per the study health facility based on the control and intervention periods in Addis Ababa, Ethiopia,** **August 2016-November 2017**

| **Variable** | | **Control/Intervention period** |  |  |  |  |
| --- | --- | --- | --- | --- | --- | --- |
|  |  |  | **Total (overall in the study period)** | **Range (in the health facilities)** | **Mean for each study health facility** | **95% CI** |
| Total U5) children involved (IMNCI & U5 contact traced) |  | Control (pre-intervention) | 85553 | 3986 (411-4398) | 1426 | 1168-1684 |
|  |  | Intervention (after intervention) | 95902 | 2862 (571-3433) | 1598 | 1439-1757 |
|  | U5 children IMNCI | Control | 85278 | 3982 (411-4393) | 1421 | 1162-1679 |
|  |  | Intervention | 95618 | 2853 (567-3420) | 1594 | 1435-1752 |
|  | U5 contacts traced | Control | 275 | 15 (0-15) | 5 | 3.9-5.3 |
|  |  | Intervention | 284 | 19 (0-19) | 4 | 3.3-5 |
| Total screened U5 (IMNC & TB DOT contacts) |  | Control | 52055 | 4266 (131-4397) | 868 | 618-1117 |
|  |  | Intervention | 93807 | 3187 (246-3433) | 1563 | 1399-1728 |
|  | U5 children screened at IMNCI | Control | 51873 | 4263 (130-4393) | 865 | 616-1113 |
|  |  | Intervention | 93570 | 3175 (245-3420) | 1560 | 1396-1723 |
|  | Contacts screened | Control | 182 | 15 (0-15) | 3 | 2.4-3.7 |
|  |  | Intervention | 237 | 18 (0-18) | 4 | 3.1-4.8 |
| Total presumptive cases |  | Control | 154 | 16 (0-16) | 3.4 | 1.3-3.4 |
|  |  | Intervention | 585 | 51 (0-51) | 10 | 7.6-12.3 |
|  | Presumptive at IMNCI | Control | 149 | 15 (0-15) | 2.3 | 1.3-3.3 |
|  |  | Intervention | 539 | 46 (0-46) | 9 | 07--11 |
|  | Presumptive TB at DOT | Control | 5 | 2 (0-2) | 0.1 | 0.0-0.2 |
|  |  | Intervention | 46 | 5 (0-5) | 0.8 | 0.5-1.02 |
| Total NGA procedures |  | Control | 18 | 9 (0-9) | 0.3 | -0.04-0.6 |
|  |  | Intervention | 107 | 20 (0-2) | 1.8 | 1-2.6 |
|  | NGA procedure at IMNCI | Control | 18 | 9 (0-9) | 0.3 | -0.04--0.6 |
|  |  | Intervention | 87 | 18 (0-18) | 1.5 | 0.8-2.2 |
|  | NGA procedure at DOT | Control | 0 | 0 | 0 | 0 |
|  |  | Intervention | 20 | 2 (0-2) | 0.3 | 0.2-0.5 |
| Total TB cases |  | Control | 11 | 4 (0-4) | 0.2 | 0.02-0.3 |
|  |  | Intervention | 48 | 4 (0-4) | 0.8 | 0.6-1.04 |
|  | TB cases at IMNCI | Control | 9 | 4 (0-4) | 0.2 | 0.0-0.3 |
|  |  | Intervention | 38 | 3 (0-3) | 0.6 | 0.4-0.8 |
|  | TB cases at DOT | Control | 2 | 1 (0-1) | 0.03 | -0.01-0.1 |
|  |  | Intervention | 10 | 2 (0-2) | 0.2 | 0.06-0.3 |
| Index cases |  | Control | 684 | 31 (5-36) | 12 | 01--01 |
|  |  | Intervention | 919 | 47(5-52) | 15 | 13-17 |
| Contact screened |  | Control | 182 |  |  |  |
|  |  | Intervention | 237 |  |  |  |
| Eligible for IPT |  | Control | 163 | 15 (0-15) | 2.8 | 2.1-3.5 |
|  |  | Intervention | 194 | 19 (0-19) | 3.3 | 2.4--4.1 |
| **Started on IPT** |  | Control | 69 | 8 (0-8) | 1.2 | 0.7-1.7 |
|  |  | Intervention | 159 | 17 (0-17) | 2.7 | 2.02-3.3 |
